# Analysis of microbial diversity in *Lagotis brevituba* Maxim. from different production areas on the Qinghai-Xizang Plateau and its correlation with secondary metabolic products

**DOI:** 10.3389/fmicb.2026.1797784

**Published:** 2026-05-05

**Authors:** Huiyuan Ma, Jianan Li, Cairen BanMa, Farong Yuan, Ying Chen, Xinyu Yang, Xue Yang, Tao Wang, Guoying Zhou

**Affiliations:** 1 Sichuan Zoige Alpine Wetland Ecosystem National Observation and Research Station, Southwest Minzu University, Chengdu, China; 2 College of Life Sciences, Qinghai Normal University, Xining, China; 3 Northwest Institute of Plateau Biology, Chinese Academy of Sciences, Xining, China; 4 Qinghai Arura Tibetan Medicine Co., Ltd., Xining, China

**Keywords:** key microbiota, *Lagotis brevituba*, microbial diversity, rhizosphere microbiota, secondary metabolites

## Abstract

*Lagotis brevituba* Maxim. (*Lagotis brevituba*), belonging to the genus *Lagotis* within the family Scrophulariaceae. The content of its secondary metabolites exhibits significant variation across different provenances, yet the underlying mechanisms linking these variations to microbial associations remain unclear. In this study, 12 samples of rhizosphere soil, root systems, and plant specimens of *Lagotis brevituba* were collected from various locations on the Qinghai-Xizang Plateau, and the physicochemical properties of the soil were systematically determined. Using high-throughput 16S and ITS rRNA sequencing technologies, the diversity and composition of bacterial and fungal communities were analyzed; simultaneously, the concentrations of 10 key secondary metabolites were quantitatively determined using high-performance liquid chromatography (HPLC). Furthermore, correlation network analysis and redundancy analysis were used to investigate the relationships among soil physicochemical factors, microbial communities, and secondary metabolites. The results indicate that an elevation of 4500 m serves as a critical threshold, marked by significant changes in soil physicochemical properties. Specifically, compared to the HA group (3500–4500 m), the EA group (elevation > 4500 m) exhibited significantly lower soil organic matter and available phosphorus content, while pH and total nitrogen content were significantly higher. These soil changes indicate the presence of nutrient-poor and alkaline stress conditions, which in turn led to the differentiation of rhizosphere bacterial communities and stimulated the synthesis of more antioxidant metabolites (e.g., significantly elevated β-Sitosterol, Quercetin, and Plantamajoside) in the EA group. Microbial community analysis revealed that bacterial community structure exhibited a significant response to changes in elevation, whereas fungal communities showed no significant differences. Soil physicochemical properties (particularly pH, SOM, and AP) are key mediating factors driving microbial community differentiation and the accumulation of secondary metabolites. A combined analysis of correlation networks and Zi-Pi values identified 19 key OTUs (Operational Taxonomic Units) mediating the relationships between microorganisms and metabolites. Notably, *Aquabacterium* exhibited significant negative correlations with Luteolin and Acteoside, while *Streptomyces* showed significant positive correlations with Hyperoside (*P* < 0.05). This study demonstrates that soil environmental factors structure microbial communities, which in turn play a crucial role in regulating the secondary metabolites of medicinal plants. These findings provide theoretical foundations for elucidating the environment-microbiome-metabolite nexus in *Lagotis brevituba*.

## 1 Introduction

Endophytic microorganisms constitute a core component of the plant microbiome, typically colonizing healthy plant tissues in bacterial or fungal forms (Compant et al., 2019; Long et al., 2025). Through co-evolution with plants, they continuously regulate growth and development, tolerance to abiotic and biotic stresses, and adaptive responses by synthesizing plant hormones, inducing systemic resistance, and secreting enzymes (Ouyang et al., 2023; Zotchev, 2024). Of particular significance, specific endophytes can form exclusive mutualistic relationships with plants, directly influencing key synthetic pathways (Li et al., 2022). This significantly alters the composition and content of endometabolites within medicinal plants, establishing them as pivotal biological factors determining the quality and yield of medicinal materials. Han et al. (2025) selected two typical chemotypes of *Atractylodes lancea* (Thunb.) to investigate the role of endophytic fungi in sesquiterpene biosynthesis. The study revealed that Nocardiaceae and other endophytic fungi within the leaves of both chemotypes activated primary metabolic pathways and signaling pathways. Wang et al. (2024) compared fungal communities in two *Lycoris* species. Their research indicates that fungi endophytes from *L. aurea* with higher galantamine content can specifically enhance the accumulation of this medicinal alkaloid in other *Lycoris* species.

The rhizosphere refers to the soil zone surrounding plant roots that is influenced by root activity. It recruits and concentrates diverse microorganisms including bacteria, fungi, forming hotspots of microbial activity and interaction in the soil near plant roots (Noman et al., 2021; Solomon et al., 2024). These microorganisms catalyze key processes such as organic matter decomposition and nutrient cycling (Bai et al., 2022), thereby laying the foundation for the formation of high-quality medicinal materials and the enrichment of secondary metabolites (Afridi et al., 2022; Mishra et al., 2022; Liu et al., 2023). Consequently, systematically analyzing the composition and diversity of rhizosphere microbial communities is crucial for elucidating plant growth and secondary metabolite production. It constitutes a prerequisite for understanding the mechanisms underlying the formation of high-quality medicinal materials and enhancing secondary metabolite accumulation.

*Lagotis brevituba*, a traditional Tibetan medicinal herb, contains chemical constituents such as flavonoids, phenylpropanoids, saponins, tannins, phenols, and polysaccharides, endowing it with significant medicinal value (Yuan et al., 2016; Li et al., 2014). It exhibits efficacy in lowering blood pressure, exhibiting antiviral and antitumor properties, and reducing blood lipids (Sun et al., 2011). It is employed in the treatment of nephritis, pulmonary diseases, hypertension, atherosclerosis, menstrual irregularities, and poisoning from various toxins (Fan et al., 2021). *Lagotis brevituba* primarily thrives in harsh environments atop high-altitude mountain scree slopes, commonly found on shady slopes of gravel belts, alpine meadows, and shrub grasslands at altitudes of 3500–4500 m (Zhang et al., 2020), exhibiting a restricted and fragmented distribution that renders it highly vulnerable (Zhu et al., 2021; Gong et al., 2022). As demand for *Lagotis brevituba* increases, excessive harvesting threatens to drastically deplete wild populations and degrade the ecological environment (Zhu et al., 2017). Therefore, the enhancement of their quality constitute the primary approaches to addressing the production constraints of *Lagotis brevituba* (Jiang et al., 2024; Duan et al., 2024). The material basis for the quality of medicinal herbs lies in their bioactive constituents, which are predominantly secondary metabolites of medicinal plants (Wang Y. et al., 2025). The biosynthetic pathways of these secondary metabolites are complex, with their synthesis and accumulation regulated by both the plant’s own genetics and various biotic and abiotic factors in the environment (Mahajan et al., 2020). This complexity impacts the quality control of medicinal materials and the development and utilization of their active components (Tanvir et al., 2024; Ogbe et al., 2020). Owing to its growth across diverse habitats on the Qinghai-Xizang Plateau, *Lagotis brevituba* develops varying adaptive strategies under the influence of different endophytic fungi and rhizosphere microorganisms. These strategies subsequently impact the plant’s growth and development processes, affecting its secondary metabolites and consequently leading to variations in its active constituents.

Concurrently, with the rapid advancement of microbiome science, researchers have progressively recognized that the microecology of traditional Chinese medicine-including endophytic bacteria within medicinal plants and rhizosphere microorganisms-directly or indirectly influences the growth (Zhou et al., 2025), metabolism, and chemical composition of medicinal materials, thereby shaping the quality of authentic medicinal herbs (Zhang X. et al., 2025; Shu et al., 2025). Extensive research indicates that secondary metabolites in medicinal plants arise from microbial activity or interactions between microbes and their hosts (Singh et al., 2022; Fan et al., 2025). Systematic investigation of the microbiome in medicinal plants can elucidate the regulatory role of microbes in secondary metabolite production, thereby contributing to enhanced medicinal plant quality.

In recent years, research on the chemical constituents of *Lagotis brevituba* has matured, with a large number of studies indicating that it primarily contains iridoid glycosides, flavonoids (such as Luteolin and Quercetin), and phenylethanoid glycosides. However, mechanistic studies specifically focusing on the microbial diversity of *Lagotis brevituba* and its association with secondary metabolites remain scarce in the published literature. Due to challenges such as the complexity of native habitat conditions, the lack of gnotobiotic models, the scarcity of culturable strain libraries, and the intricate interactions among multi-species microbiota coupled with the absence of systematic models, there is a lack of comprehensive research on the relationship between the microbiome and secondary metabolites in *Lagotis brevituba*. Therefore, this study selected *Lagotis brevituba* from different production areas as the research subject. Through extensive sample collection, high-throughput sequencing of 16S and ITS rRNA was employed to analyze microbial community composition and diversity. Simultaneously, high-performance liquid chromatography (HPLC) was utilized for quantitative analysis of secondary metabolite content. This study investigates the species composition, diversity, and co-occurrence networks of endophytic bacteria in *Lagotis brevituba* roots and rhizosphere soil microbial communities. It further identifies key microbial communities and their relationships with secondary metabolites, thereby comprehensively analyzing how microbial communities influence the active compounds in *Lagotis brevituba* from different regions. The research aims to elucidate (i) differences in soil physicochemical properties and secondary metabolites among *Lagotis brevituba* from various regions; (ii) the composition and diversity of endophytic bacteria in *Lagotis brevituba* roots and rhizosphere soil microorganisms; (iii) the correlation between microorganisms and secondary metabolites in *Lagotis brevituba*. The findings will provide a theoretical basis for enhancing the quality of active constituents in *Lagotis brevituba* as a medicinal plant and addressing related microecological issues. It will also furnish scientific data for the conservation of *Lagotis brevituba* resources.

## 2 Materials and methods

### 2.1 Sample collection and processing

In 2024, samples of *Lagotis brevituba* were collected from 12 sites across Qinghai Province, Sichuan Province, and the Tibet Autonomous Region. At each sampling location, 10 plants were randomly selected for sampling. The longitude, latitude, and altitude of each sampling site were recorded using a Global Positioning System. The sampling range spanned longitudes from 95°E to 101°E, latitudes from 31°N to 38°N, and altitudes from 3500 to 4800 m. Specimens were collected from alpine scree slopes and upper subalpine meadows. Sampling plots were selected using random sampling methods, typically comprising 3 m × 3 m squares, with adjustments made to individual plots based on practical considerations. At each site, ten medicinal plant specimens were randomly collected. Using a sterilized spade, a pit measuring 0.3 m wide × 0.3 m long × 0.5 m deep was excavated to remove the plants intact from their roots. Combine the samples into a composite sample, place it in a sterile bag, and assign a reference number. Transport the collected plant material to the laboratory. Wash the roots and surface soil residue from one portion, then immerse in distilled water for 12 h. Separate the roots from the leaves. Thoroughly wash the roots repeatedly to remove surface soil, perform sterile processing, place in test tubes, and store at −80°C for metabolomic and microbiological analysis. The remaining portion was air-dried naturally, weighed, and recorded. The entire plant was then pulverized, passed through a 65-mesh sieve, and subsequently subjected to high-performance liquid chromatography to determine the content of active constituents. All plant samples were identified by Professor Guoying Zhou of the Northwest Plateau Institute of Biology, and the specimens were stored in the herbarium of the research unit.

### 2.2 Determination of soil physicochemical properties for *Lagotis brevituba*

Soil samples from the rhizosphere were placed in a vehicle-mounted refrigerator and transported back to the laboratory for further processing. Upon return to the laboratory, the soil samples were air-dried. Once dried, the rhizosphere soil was sieved through a 65-mesh screen, thoroughly mixed, and stored in a −80°C freezer. Soil physicochemical properties included soil pH, total nitrogen (TN), total phosphorus (TP), available phosphorus (AP), soil organic matter (SOM), ammonium nitrogen (NH_4_^+^-N), and nitrate nitrogen (NO_3_^–^-N). Testing methods followed the third edition of Soil Agrochemical Analysis (China Agricultural Press, 2000): Soil pH was determined using the pH meter potential method (water-to-soil ratio 2.5:1); soil total nitrogen was measured by the Kjeldahl method; total phosphorus by the sulfuric acid-perchloric acid digestion molybdenum-antimony-barium colorimetric method; available phosphorus by sodium bicarbonate extraction followed by the molybdenum-antimony-barium colorimetric method; organic matter content was determined using the potassium dichromate external heating method; soil ammonium nitrogen and nitrate nitrogen were extracted with potassium chloride solution and analyzed using an automatic discontinuous chemical analyzer.

### 2.3 Quantitative analysis of metabolites in *Lagotis brevituba*

The whole plants of *Lagotis brevituba* collected from various origins were sun-dried, pulverized, and sieved through a 65-mesh screen. Content analysis was conducted using an Agilent 1260 High Performance Liquid Chromatography (HPLC) system (Agilent Technologies) with a Unitary C18 column (4.6 nm × 250 nm, 5 μm, 100 A) supplied by Huapu Xinchuang Technology Co., Ltd., Chromatographic-grade methanol and acetonitrile were procured from Yu Wang Group (Shandong, China). Metabolite reference standards employed for quantification: Echinacoside (82854-37-3), Plantamajoside (104777-68-6), Acteoside (61276-17-3), Hyperoside (482-36-0), Luteolin (491-70-3), Cynaroside (5373-11-5), Apigenin-7-glucoside (578-74-5), β-Sitosterol (83-46-50), Quercetin (117-39-5), and Apigenin (520-36-5) were all procured from Chengdu Desite Biotechnology Co., Ltd., (Sichuan, China).

Referring to the experimental protocol of the Chinese Pharmacopoeia Commission Chinese Pharmacopoeia (2020), Xing et al. (2012) and Shi et al. (2003). We determined a total of ten secondary metabolites: Echinacoside, Plantamajoside, Acteoside, Hyperoside, Quercetin, Beta-Sitosterol, Luteolin, Cynaroside, Apigenin 7-O-β-glucoside and Apigenin. Three precise 0.5 g powder samples were dissolved in 25 mL 80% methanol within conical flasks, subjected to ultrasonic treatment for 30 min (40°C), then allowed to settle. Subsequently, the solution was filtered through a 0.22 μm membrane filter and transferred to a liquid chromatography vial for future use. Standard samples were employed to prepare a standard stock solution. Prepared standard samples and test samples were analyzed concurrently on the instrument. Finally, the content of active constituents in *Lagotis brevituba* was calculated based on retention time and peak area. The HPLC conditions are as follows: (1) Echinacoside, Plantamajoside, and Acteoside: the mobile phase consists of acetonitrile (A) and 0.1% phosphoric acid in water (B). The elution gradient is as follows: 0–40 min (12%–40% A; 88%–60% B); flow rate 1.0 mL/min; column temperature 25°C; detection wavelengths 254, 330, 360 nm; injection volume 20.00 μL. (2) Hyperoside, β-Sitosterol, Quercetin: mobile phase: acetonitrile (A) and 0.2% phosphoric acid in water (B). Elution gradient: 0–12 min (19% A, 81% B); 12–20 min (19%–55% A, 81%–45% B); 20–29 min (55%–75% A, 45%–25% B); 29–40 min (75%–85% A, 25%–15% B); 40–49 min (85%–90%, A; 15%–10%, B); 49–60 min (90%–12%, A; 10%–88%, B); flow rate 1.0 mL/min; column temperature 25°C; detection wavelengths 205 and 283 nm; injection volume 20.00 μL. (3) Luteolin: the mobile phase consists of 0.4% formic acid in water (A) and acetonitrile (B). The elution gradient is as follows: 0–16 min (86%–76%, A; 14%–24%, B); 16–20 min (76%–60%, A; 24%–40%, B); 20–23 min (60%–40% A, 40%–60% B); 23–26 min (40%–10% A, 60%–90% B); 26–31 min (10%–5%, A; 90%–95%, B); flow rate 1.0 mL/min; column temperature 35°C; detection wavelengths 336, 348, 350 nm; injection volume 20.00 μL. (4) Cynaroside, Apigenin, and Apigenin-7-O-β-D-glucoside: the mobile phase consists of acetonitrile (A) and 0.1% phosphoric acid aqueous solution (B). The elution gradient is as follows: 0–20 min (16% A; 84% B); 20–22 min (16%–20% A, 84%–80% B); 22–25 min (20%–25% A, 80%–75% B); 25–30 min (25%–35% A, 75%–65% B); 30–35 min (35%–90%, A; 65%–10%, B); 35–40 min (90%–16%, A; 10%–84%, B); flow rate 1.0 mL/min; column temperature 35°C; detection wavelengths 330, 336, 348 nm; injection volume 20.00 μL.

### 2.4 DNA extraction and high-throughput sequencing of endophytic fungi from *Lagotis brevituba*

Weigh 0.10–0.17 g of plant samples and 0.23–0.30 g of soil samples, for a total of 24 samples, to complete the DNA extraction from soil and plant endophytic bacteria samples. Following the manufacturer’s protocol, total genomic DNA from microbial communities was extracted from soil and plant samples using the FastPure Soil DNA Isolation Kit. The quality of extracted genomic DNA was assessed via 1% agarose gel electrophoresis, whilst DNA purity and concentration were quantified spectrophotometrically using the NanoDrop2000 (Thermo Scientific, USA). The V5-V7 region of the bacterium was amplified using primer pairs 799F-1392R and 799F-1193R. The ITS1 region of the fungi was amplified using the primers ITS1F-ITS2R. Microbial community high-throughput sequencing was performed by Shanghai Majorbio Biotechnology Co., Ltd., Raw sequences underwent quality control processing in Qiime (v1.91) and Uparse (v11), followed by operational taxonomic unit (OTU) clustering at 97% similarity and chimera removal using UPARSE software (Anderson et al., 2021)^1^. Chimeric sequences were identified and filtered using Mothur (v1.30.2). OTU species taxonomic annotation was performed with the RDP Classifier (v11.5) at a confidence threshold of 70%. Following rarefaction and removal of OTUs associated with chloroplasts and mitochondria, the final dataset comprised 8,922 bacterial OTUs and 3,827 fungi OTUs. To assess sequencing saturation, rarefaction curves were generated based on observed OTUs. All curves plateaued at the current sequencing depth, indicating that the majority of microbial diversity was captured in each sample (Supplementary Figures 1, 2). All sequencing data generated or analyzed during this study have been submitted to the NCBI Sequence Read Archive (SRA) database under accession numbers PRJNA1448557 and PRJNA1448718.

### 2.5 Statistical analysis of data

ANOVA (Analysis of variance) was employed to examine the soil physicochemical properties and secondary metabolite content of *Lagotis brevituba* from different provenances. Alpha diversity metrics including Chao 1 and Shannon indices were calculated using Mothur software,^2^ with inter-group differences analyzed (Chao and Bunge, 2002). Additionally, PCoA analysis (Principal Co-ordinates Analysis) based on Bray–Curtis distance was conducted to demonstrate microbial community β-diversity, and ADONIS (PERmutational Multivariate ANalysis Of Variance) tests were performed to analyze the effects of different provenances on the microflora of *Lagotis brevituba*. Microbial symbiotic networks were constructed using the igraph package and Gephi software. The Spearman correlation matrix was thresholded at *P* < 0.05 and R ≥ 0.5. Zi-Pi values were calculated across the network to identify key bacterial groups. Key microorganisms were selected based on Spearman’s correlation analysis for correlation network mapping, including analysis of correlations with secondary metabolites, to screen microorganisms associated with the content of secondary metabolites in *Lagotis brevituba* (Zhou et al., 2017).

## 3 Results

### 3.1 Analysis of soil physicochemical properties and secondary metabolites in *Lagotis brevituba*

**FIGURE 1:**
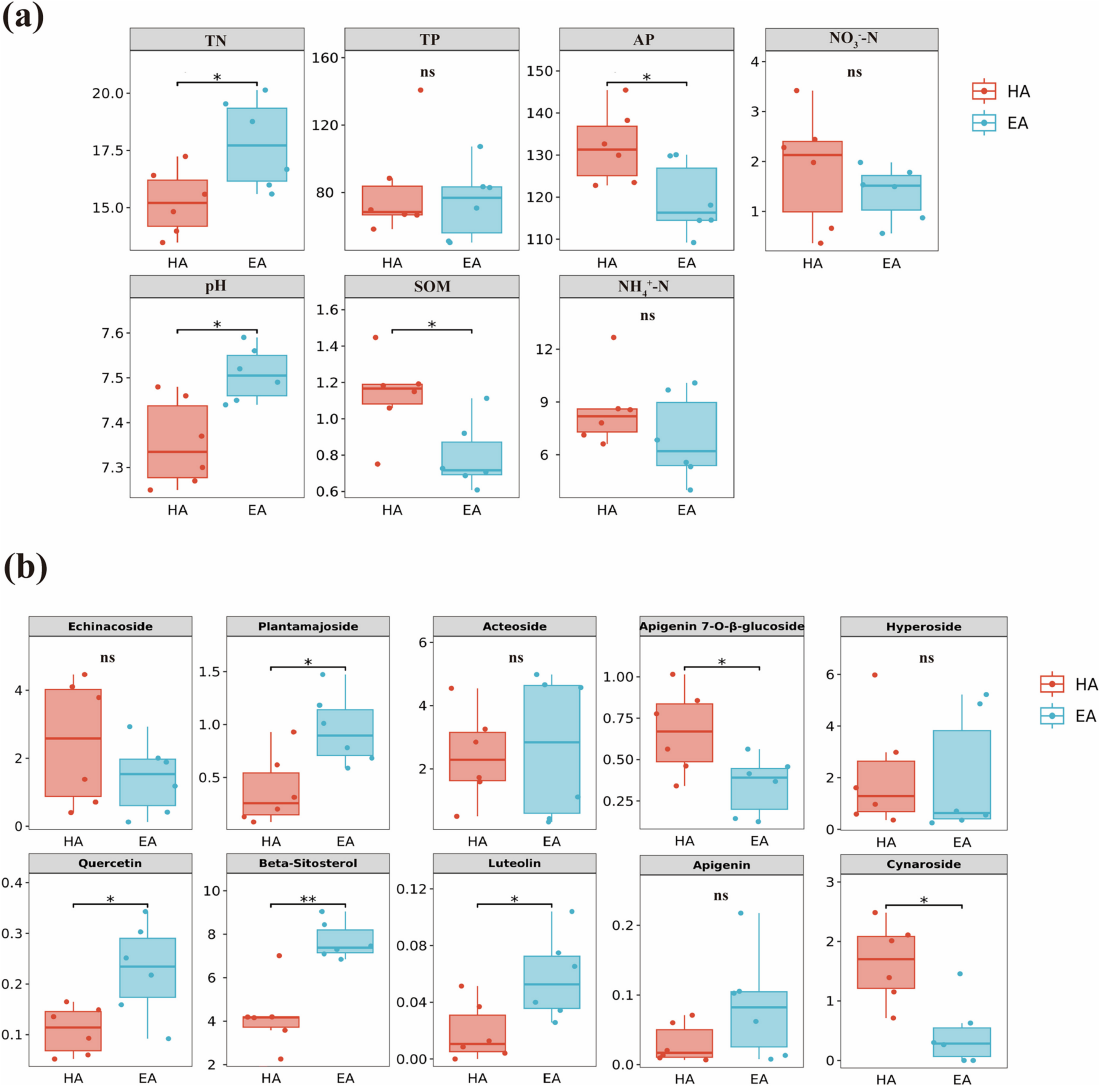
**(a)** Differences in soil physicochemical properties between HA and EA groups of *Lagotis brevituba*. **(b)** Differential metabolite profile of HA and EA groups in *Lagotis brevituba*. The significance levels are as follows: *P* < 0.05, one asterisk (*); *P* < 0.01, two asterisks (**); *P* < 0.001, three asterisks (***).

Seven parameters–total nitrogen (TN), total phosphorus (TP), available phosphorus (AP), nitrate nitrogen (NO_3_^–^-N), pH, ammonium nitrogen (NH_4_^+^-N), and soil organic matter (SOM)–were measured in the rhizosphere soils of *Lagotis brevituba* from different locations. Through data analysis, we identified a significant altitudinal boundary at 4500 m, dividing the *Lagotis brevituba* samples into two distinct groups: HA group (samples collected between 3500 and 4500 m elevation) EA group (samples collected above 4500 m elevation) (Table 1). Results indicate that soil physicochemical properties exhibit significant variations with increasing altitude across different locations. As shown in Figure 1a, significant differences were observed in soil organic matter (SOM), total nitrogen (TN), available phosphorus (AP), and pH, SOM and AP in the EA group were significantly lower than in the HA group (*P* < 0.05), whereas TN and pH showed the opposite trend. This indicates that altitudes below 4500 m are more conducive to the accumulation of soil organic matter and available phosphorus. There were no significant differences in TP (total phosphorus), NO_3_^–^-N (nitrate nitrogen), or NH_4_^+^-N (ammonium nitrogen). Within this, the HA group exhibited slightly higher NO_3_^–^-N than the EA group, while the opposite was true for NH_4_^+^-N. Ammonium nitrogen and nitrate nitrogen concentrations were relatively low with no significant differences.

**TABLE 1:** Specific sampling information and grouping details for *Lagotis brevituba*.

No.	Altitude (m)	Longitude	Latitude	Group
LB1	3920	101°23′38.45′′	37°21′0.14′′	HA
LB2	3962	98°52′31.41′′	37°10′32.08′′	HA
LB3	3566	100°16′0.25′′	38°5′30.59′′	HA
LB4	4078	100°14′23.05′′	38°0′53.15′′	HA
LB5	4497	99°30′59.70′′	35°29′54.14′′	HA
LB6	4364	99°18′51.08′′	35°24′6.10′′	HA
LB7	4571	96°28′21.10′′	31°57′13.23′′	EA
LB8	4729	95°11′3.44′′	32°57′49.98′′	EA
LB9	4660	95°52′56.58′′	32°56′39.14′′	EA
LB10	4839	97°12′44.92′′	32°34′14.55′′	EA
LB11	4733	98°50′21.73′′	31°47′10.31′′	EA
LB12	4629	96°40′44.98′′	31°23′58.55′′	EA

Through HPLC, we determined the content of ten secondary metabolites: Echinacoside, Plantamajoside, Acteoside, Hyperoside, Quercetin, β-Sitosterol, Luteolin, Cynaroside, Apigenin 7-O-β-glucoside and Apigenin. As shown in Figure 1b, Plantamajoside, Luteolin, and Quercetin were significantly higher in the EA group than in the HA group (*P* < 0.05), whilst β-Sitosterol was significantly higher in the EA group than in the HA group (*P* < 0.01). Conversely, the HA group exhibited significantly higher levels of Apigenin 7-O-β-glucoside and Cynaroside than the EA group (*P* < 0.05). Furthermore, although no significant difference was observed in Echinacoside content, the HA group exhibited higher levels. Acteoside, Hyperoside, and Apigenin showed no significant differences, yet the EA group contained slightly higher amounts than the HA group. These results indicate that most compounds were significantly more abundant in the EA group than in the HA group, suggesting that higher altitudes may confer greater biological activity or enhanced antioxidant capacity.

**FIGURE 2:**
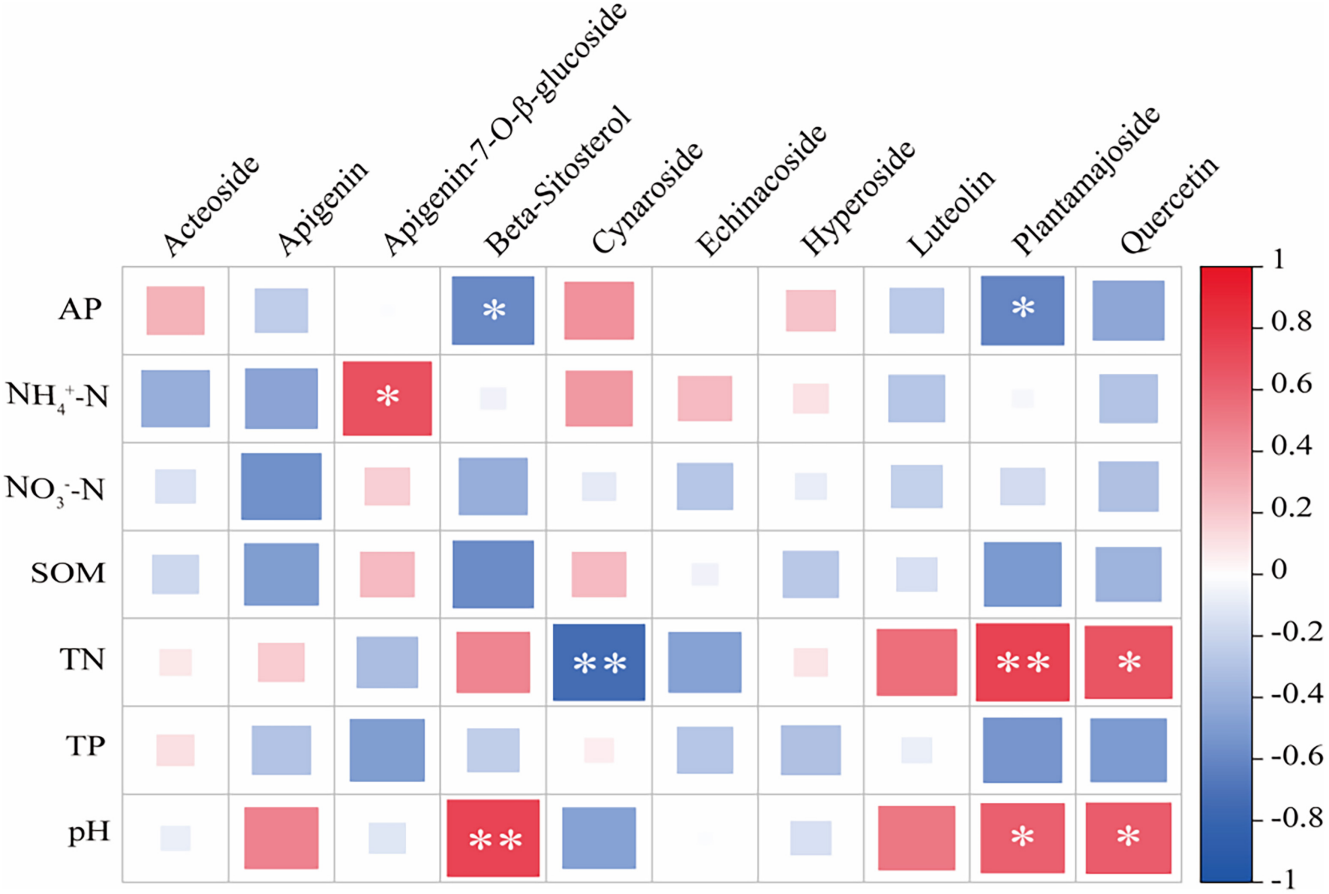
Correlation map of soil factors and active ingredient content in *Lagotis brevituba*. The significance levels are as follows: *P* < 0.05, one asterisk (*); *P* < 0.01, two asterisks (**); *P* < 0.001, three asterisks (***).

The correlations between soil factors and secondary metabolites of *Lagotis brevituba* are shown in the Figure 2. Available phosphorus (AP) was significantly negatively correlated with β-Sitosterol and Plantamajoside; ammonium nitrogen (NH_4_^+^-N) showed a significant positive correlation with Apigenin-7-O-β-D-glucoside; total nitrogen (TN) showed a significant negative correlation with Luteolin, and a significant positive correlation with Plantamajoside and Quercetin; pH was significantly positively correlated with β-Sitosterol, Plantamajoside, and Quercetin.

In this study, altitude was positively correlated with the levels of β-Sitosterol, Quercetin, and Plantamajoside, and these antioxidant compounds were synergistically enriched in the EA group (Figure 1b). Soil-metabolite correlation analysis (Figure 2) further supports this association: pH showed a significant positive correlation with β-Sitosterol, Plantamajoside, and Quercetin, while available phosphorus (AP) showed a significant negative correlation with β-Sitosterol and Plantamajoside, directly quantifying the statistical association between soil alkalization and oligotrophic conditions and the accumulation of antioxidant metabolites.

### 3.2 Analysis of microbial community alpha and beta diversity

**FIGURE 3:**
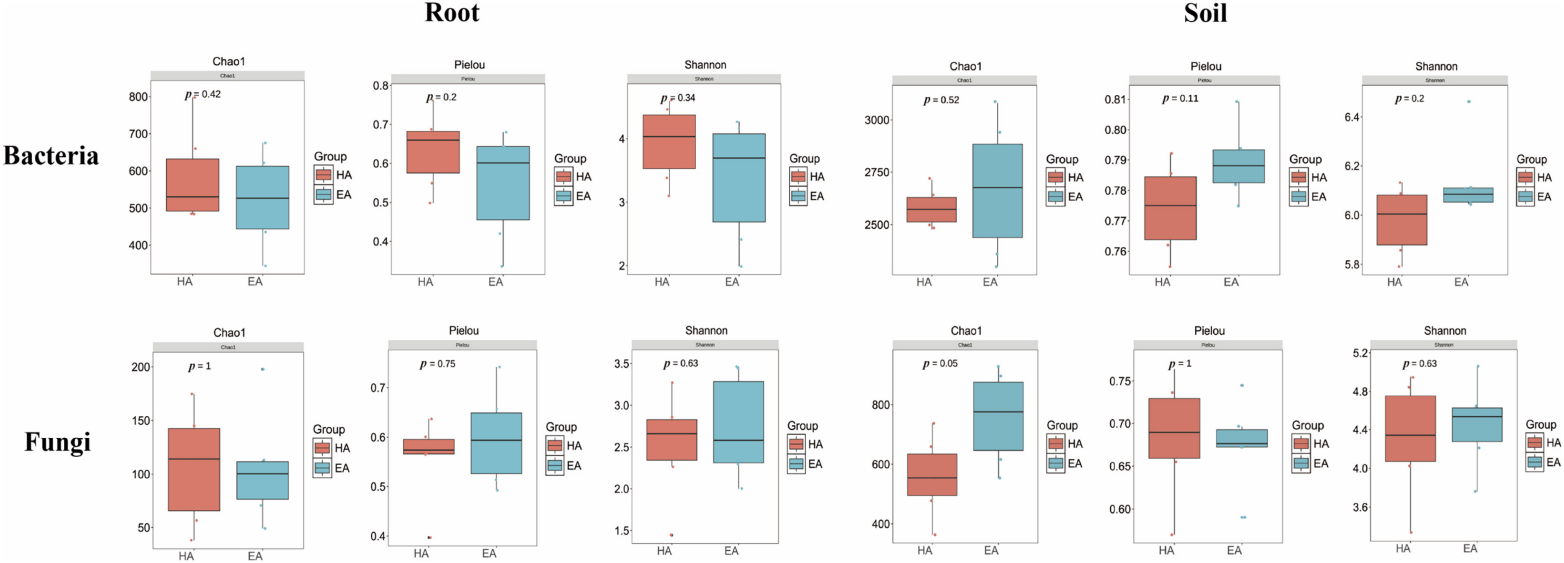
Differences in alpha diversity between HA and EA of *Lagotis brevituba*.

The Wilcoxon test was employed to compare root-associated endophytic bacteria and fungi, as well as soil microbial alpha diversity indices (Shannon, Pielou, and Chao1) between the HA and EA groups. Results indicated no significant differences in alpha diversity indices between the two groups (Figure 3). Specifically, the HA group exhibited higher Chao1, Shannon, and Pielou indices for both bacteria and fungi than the EA group, though these differences failed to reach statistical significance. Analysis of soil microbial alpha diversity revealed no significant differences between the HA and EA groups in bacterial alpha diversity indices. However, for fungi alpha diversity indices, the Chao1 index indicated a significant difference between groups (*P* = 0.05), whereas the Pielou and Shannon indices showed no significant differences.

**FIGURE 4:**
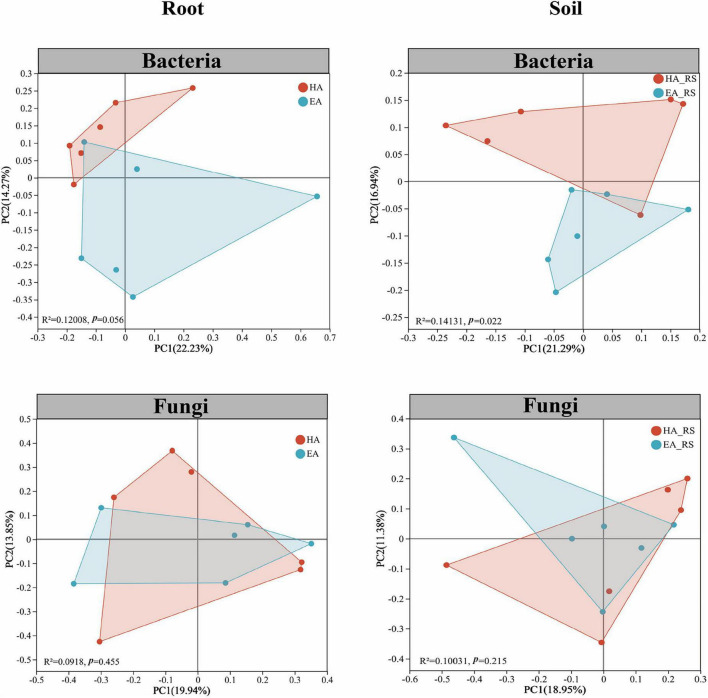
PCoA based on the Bray-Curtis distance matrix revealed similarities or differences in community composition between root-associated microbiomes and rhizosphere soil microorganisms.

To explore differences between the root-associated microbiome and rhizosphere soil microbiota, we performed PCoA using the Bray-Curtis distance matrix. PCoA revealed similarities or differences in community composition between root-associated microbiomes and rhizosphere soil microbiota. PCoA analysis showed that bacterial communities in the HA and EA groups were significantly separated, forming distinct clustering clusters (Figure 4). In contrast, fungal communities exhibited no significant structural differences between HA and EA groups. Specifically, for endophytic bacteria, ADONIS analysis indicated significant structural differences in bacterial communities between HA and EA groups (*R*^2^ = 0.12008, *P* = 0.056), with PC1 explaining 22.23% and PC2 explaining 14.27% of the variance. In rhizosphere soil microorganisms, bacterial community structure differed significantly between HA and EA groups (*R*^2^ = 0.14131, *P* = 0.022), with PC1 explaining 21.29% and PC2 explaining 16.04% of the variation. For endophytic fungi, ADONIS analysis indicated no significant differences in fungal community structure between HA and EA groups (*R*^2^ = 0.0918, *P* = 0.455), with PC1 explaining 19.94% and PC2 explaining 13.85% of the variance. Among rhizosphere soil microorganisms, fungal community structure differed non-significantly between HA and EA groups (*R*^2^ = 0.10031, *P* = 0.215), with PC1 explaining 18.95% and PC2 explaining 11.38% of the variance. Overall, our findings indicate that bacterial community structure exhibits greater variation than fungal community structure, particularly within rhizosphere soils. Furthermore, rhizosphere soil microorganisms demonstrate heightened sensitivity to altitude changes compared to endophytic microorganisms, with rhizosphere bacterial communities being especially responsive.

### 3.3 Analysis of microbial community composition

**FIGURE 5:**
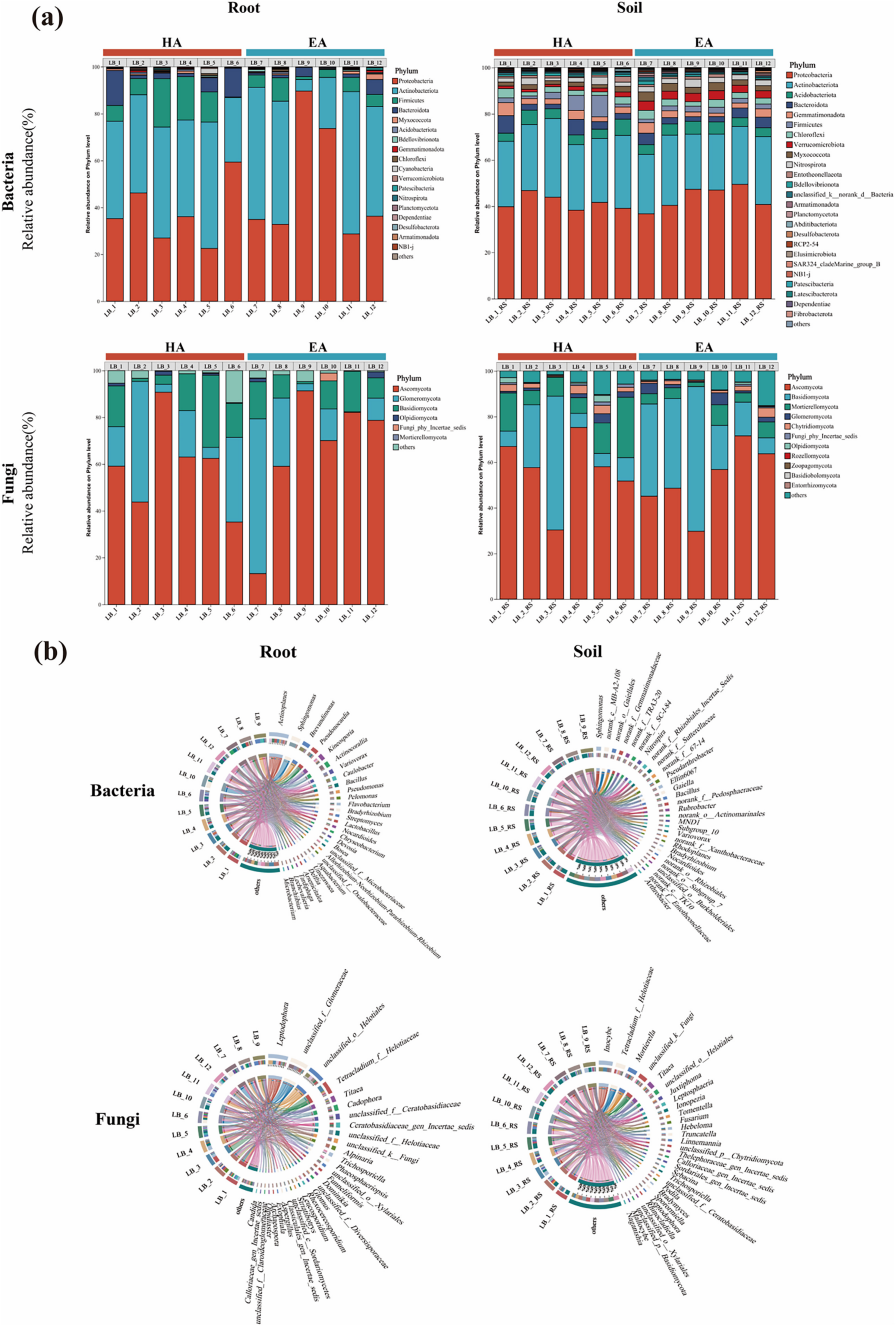
**(a)** *Lagotis brevituba* root system and soil microbial species composition at phylum level. **(b)** *Lagotis brevituba* root system and soil microbial species composition at genus level.

A total of 1,666,629 optimized bacterial sequences and 3,045,074 optimized fungal sequences were obtained from 16S rRNA and ITS regions respectively. Following data quality control and removal of plant sequences, 8,922 bacterial OTUs and 3,827 fungal OTUs were obtained across all samples. As depicted in Figure 5a, at the phylum level, the primary dominant bacterial phyla within root endophytes were Proteobacteria, with Actinobacteria and Firmicutes as secondary dominant phyla. Proteobacteria exhibited absolute dominance across all samples, with relative abundances generally ranging from 40% to 60%. Notably, Proteobacteria relative abundance was significantly higher in the HA group than in the EA group. Ascomycota constituted the primary dominant fungi phylum, with relative abundances typically ranging from 60% to 80%. Glomeromycota and Basidiomycota constituted the secondary dominant fungi phyla. The primary dominant bacterial phyla in rhizosphere soil microbiota were Proteobacteria and Actinobacteria. Compared to root samples, the relative abundance of Proteobacteria in soil was comparatively reduced. In soil samples, Ascomycota remained the dominant fungi phylum but exhibited significantly reduced abundance (most samples <50%). Basidiomycota and Mortierellomycota constituted secondary dominant phyla. Compared to root samples, soil displayed higher phylum-level fungi diversity and more pronounced compositional variation between samples. From this, we conclude that the root microbial community exhibited a higher proportion of dominant phyla but lower diversity. Dominant bacterial/fungi phyla in roots generally accounted for >50%, whereas soil communities showed greater dispersion.

To gain further insight into the microbial community structure within *Lagotis brevituba*, we employed a Circos sample-species relationship diagram. The inner colored bands connect species, thereby illustrating their abundance distribution across samples. At the genus level, as depicted in Figure 5b, the most abundant endophytic bacterial group within roots was *Actinoplanes*, followed by *Sphingomonas*. Among abundant fungi genera, *Leptodophora* exhibited the highest abundance in root samples, with unclassified_f__Glomeraceae ranking second. The soil microbial Circos diagram indicates *Sphingomonas* as a key bacterial group. Notably, *Bradyrhizobium* exhibits higher abundance in LB-1, suggesting its nitrogen-fixing potential. Inocybe was the most abundant fungi genus, with *Mortierella*, unclassified_k__Fungi, and *Tetracladium*_f__Helotiaceae as secondary fungi genera.

### 3.4 Co-occurrence network analysis of *Lagotis brevituba* and key microbial communities

**FIGURE 6:**
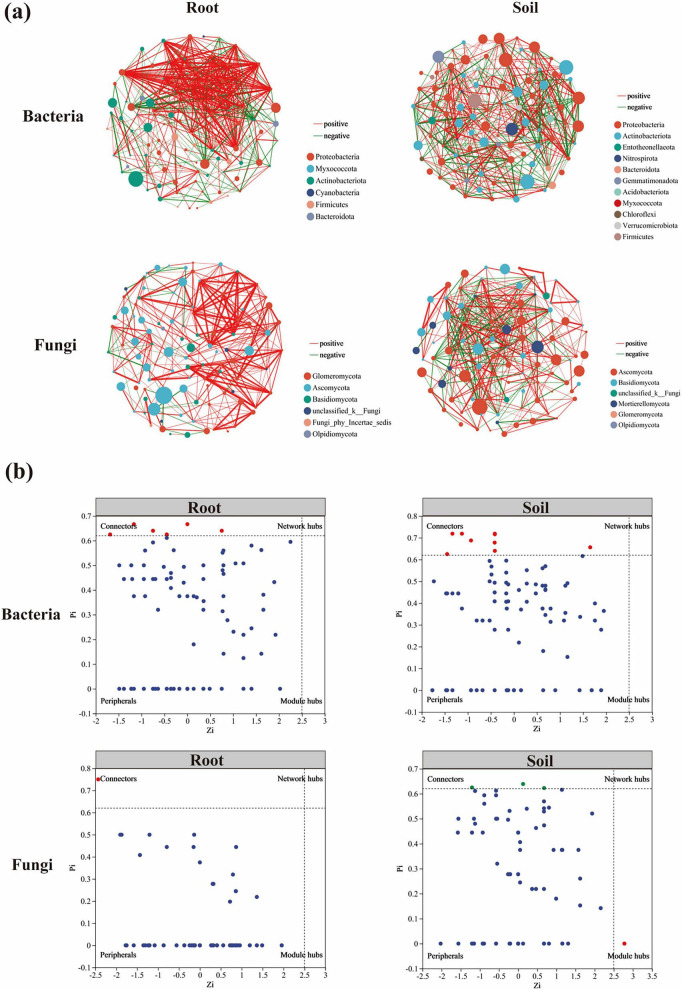
**(a)** Correlation network diagram of endophytic bacteria and fungi within the roots of *Lagotis brevituba*, and rhizosphere soil bacteria and fungi. The red lines in the diagram denote positive correlations, while the green lines indicate negative correlations; the thickness of the lines represents the magnitude of the correlation coefficients. **(b)** Computing the intra-module connectivity (Zi) and inter-module connectivity (Pi) of each node based on co-occurrence networks.

Based on Spearman’s correlation analysis, correlation networks were constructed for endophytic bacteria and fungi within the roots of *Lagotis brevituba*, as well as for soil bacteria and fungi in the rhizosphere. As shown in Figure 6a, analysis of the root endophytic bacterial network revealed 426 positive correlations and 142 negative correlations at the OTU level, with an average degree of 11.47 and a clustering coefficient of 0.388. The rhizosphere soil bacterial network exhibited 303 positive correlations and 243 negative correlations, with an average degree of 10.92 and a clustering coefficient of 0.379. Both the root-associated bacterial network and the rhizosphere soil bacterial network predominantly featured Proteobacteria nodes, alongside Actinobacteria. The root-associated fungal network comprised 283 positive correlations and 36 negative correlations, with an average degree of 6.38 and a clustering coefficient of 0.450. Nodes predominantly belonged to Glomeromycota and Ascomycota. The rhizosphere soil fungi network exhibited 271 positive correlations and 163 negative correlations, with an average degree of 8.86 and a clustering coefficient of 0.426. Nodes in this network were predominantly represented by Ascomycota and Basidiomycota.

This study employed the Zi-Pi network topology analysis method to identify key microbial groups. Specifically, after constructing a microbial co-occurrence network based on Spearman’s correlation, the intra-module connectivity (Zi) and inter-module connectivity (Pi) of each node were calculated. The classification criteria are as follows: module hubs (Zi > 2.5 and Pi < 0.62) and connectors (Zi < 2.5 and Pi > 0.62) and network hubs (Zi > 2.5 and Pi > 0.62) are collectively defined as keystone microbiota, i.e., the “key microbiota” in the context of this study. Such nodes occupy central topological positions within the network and play a crucial role in maintaining community structure and function. Based on the above classification criteria, by Zi-Pi calculations, 19 key nodes were identified, belonging to seven phyla (Figure 6b). Specifically, six highly connected nodes were identified within the root-associated bacterial network, predominantly belonging to the Actinobacteriota phylum and Proteobacteria phylum. Within the root-associated fungi network, only one highly connected node was identified, belonging to the Ascomycota phylum. Within the rhizosphere soil bacterial network, eight highly connected nodes were identified, predominantly from the Actinobacteriota and Proteobacteria phyla, with two individual nodes belonging to Acidobacteriota and Gemmatimonadota respectively. Three highly connected nodes were identified in the rhizosphere soil fungi network, belonging to the Basidiomycota and Mortierellomycota phyla respectively. Notably, a network hub node with high connectivity across the entire network was also identified in the rhizosphere soil fungi network, belonging to the Ascomycota phylum.

### 3.5 Correlation analysis of soil physicochemical properties and secondary metabolites in *Lagotis brevituba*

**FIGURE 7:**
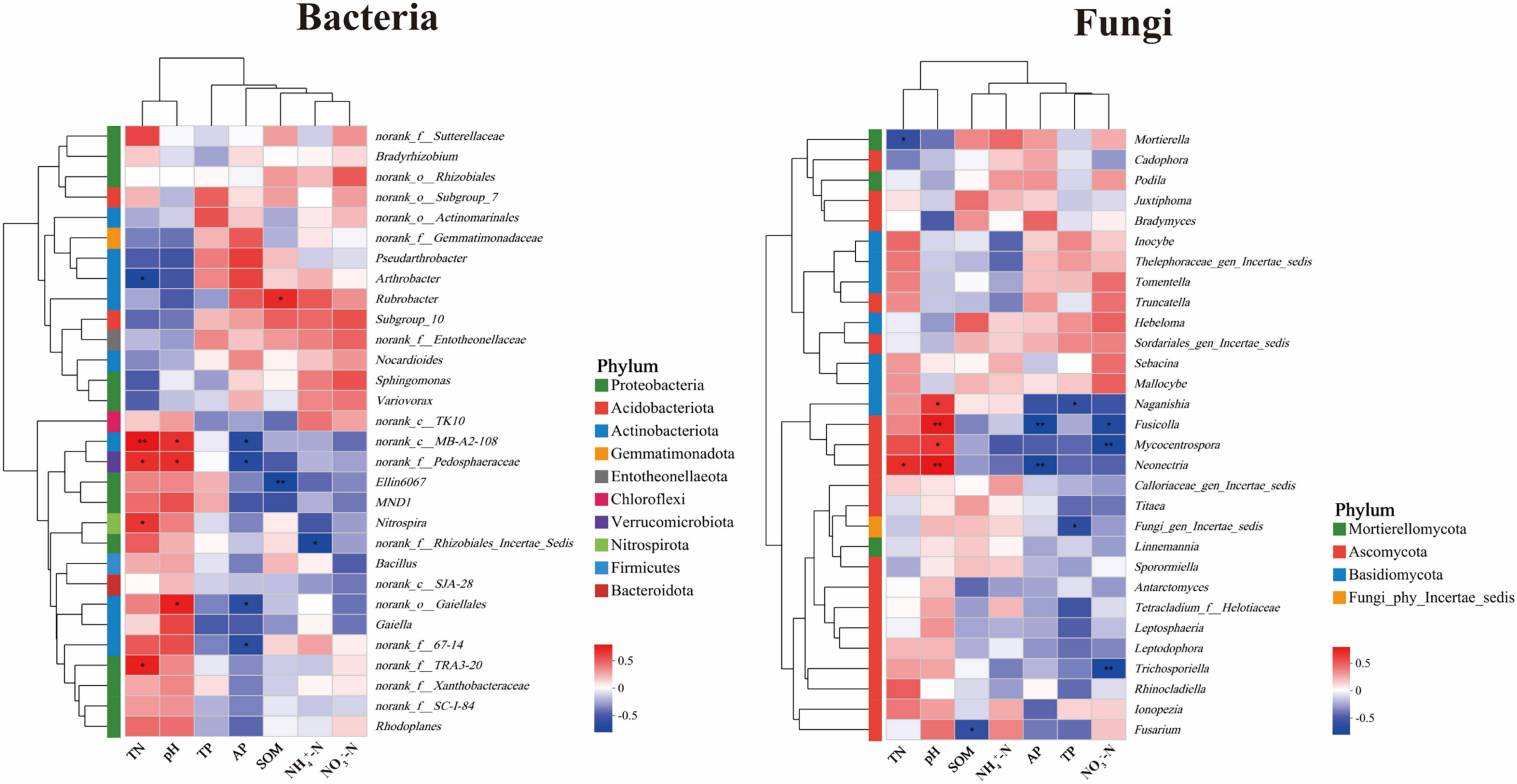
Heatmap of genus-level correlations between microbial communities and soil environmental factors. The significance levels are as follows: *P* < 0.05, one asterisk (*); *P* < 0.01, two asterisks (**); *P* < 0.001, three asterisks (***). Coefficients without significance are not shown.

Through correlation heatmap analysis, the relationship between the top 30 microbial groups by total abundance in the rhizosphere soil of *Lagotis brevituba* and soil physicochemical indicators was examined. Results indicate, as shown in Figure 7, that factors such as pH, available phosphorus (AP), total nitrogen (TN), and soil organic matter (SOM) exert a significant influence on the dominant microbial groups within the rhizosphere soil. Specifically, for bacteria, the dominant microbial groups in rhizosphere soil predominantly belonged to Proteobacteria, followed by Actinobacteria, consistent with previous findings on dominant bacterial community species composition. norank_c__MB-A2-108 exhibited a significant positive correlation with TN (*P* < 0.01), a significant positive correlation with pH, and a significant negative correlation with AP (*P* < 0.05). Ellin6067 exhibited a significant negative correlation with SOM (*P* < 0.01). Nitrospira showed a significant positive correlation with TN, consistent with its role in nitrogen cycling. norank_f__Pedosphaeraceae demonstrated significant positive correlations with TN and pH, and a significant negative correlation with AP (*P* < 0.05). Rubrobacter exhibited a significant positive correlation with SOM (*P* < 0.05). norank_o__Gaiellales showed a significant positive correlation with pH and a significant negative correlation with AP (*P* < 0.05). For fungi, the dominant microbial groups in the rhizosphere soil predominantly belong to the Ascomycota, followed by the Basidiomycota and Mortierellomycota, consistent with the results of the dominant fungi community species composition mentioned earlier. *Fusicolla* exhibited a significant positive correlation with pH (*P* < 0.01), a significant negative correlation with AP (*P* < 0.01), and a significant negative correlation with NO_3_^–^-N. *Naganishia* exhibited a significant positive correlation with pH and a significant negative correlation with TP (*P* < 0.05). *Neonectria* showed significant positive correlations with TN and pH, and a significant negative correlation with AP (*P* < 0.01). *Trichosporiella* exhibited a significant negative correlation with NO_3_^–^-N (*P* < 0.01). *Mycocentrospora* showed a significant positive correlation with pH and a significant negative correlation with NO_3_^–^-N (*P* < 0.01).

**FIGURE 8:**
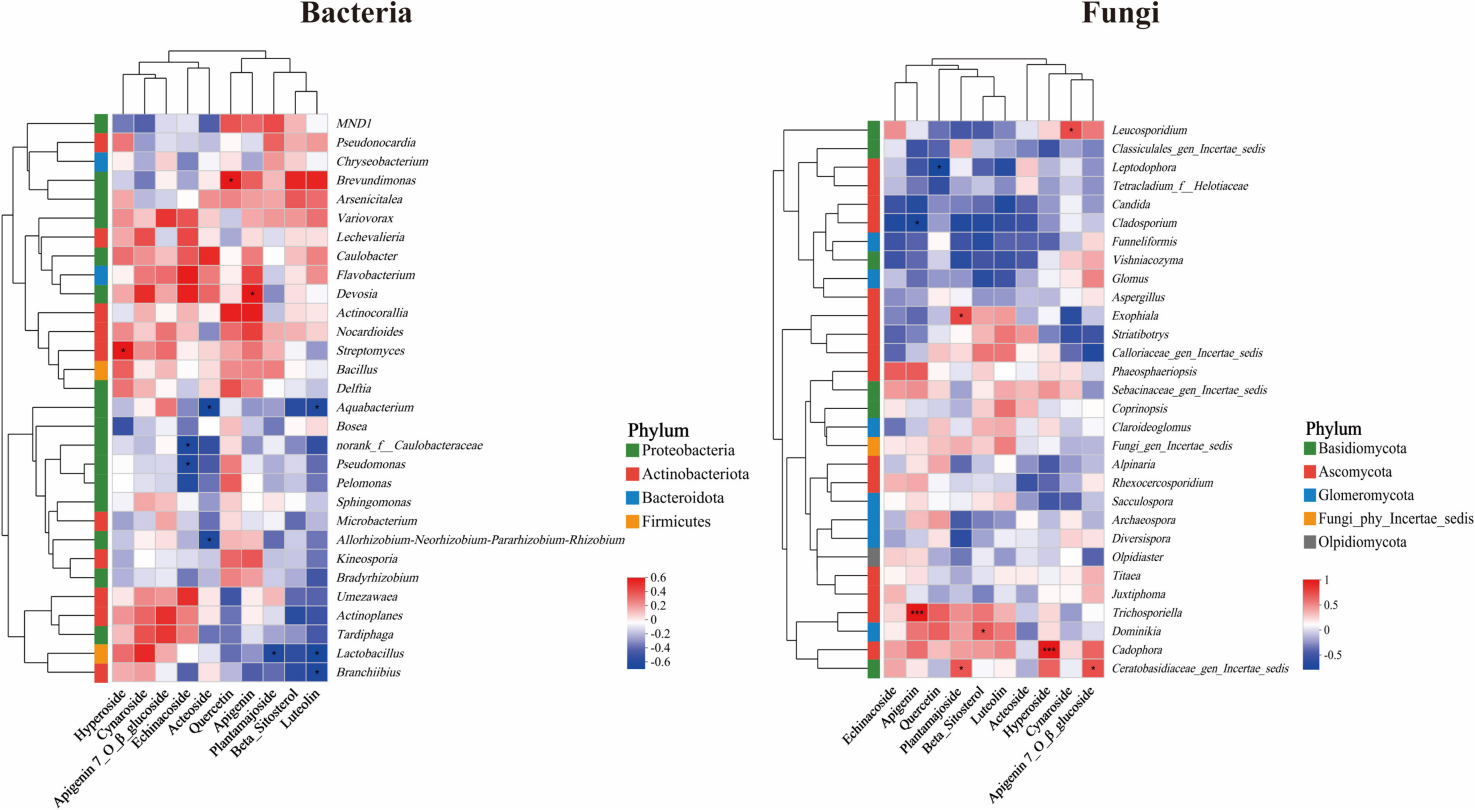
Heatmap of correlations between microbial communities and secondary metabolites. The significance levels are as follows: *P* < 0.05, one asterisk (*); *P* < 0.01, two asterisks (**); *P* < 0.001, three asterisks (***). Coefficients without significance are not shown.

Through correlation heatmap analysis, the relationship between the top 30 microbial taxa by total abundance within the roots of *Lagotis brevituba* and secondary metabolites was examined. The analysis revealed, as shown in Figure 8, that for bacteria, the dominant endophytic microbial groups predominantly belonged to the Proteobacteria phylum, with Actinobacteria being the secondary dominant phylum, consistent with the previous findings on the species composition of dominant bacterial communities. *Aquabacterium* exhibited significant negative correlations (*P* < 0.05) with Luteolin and Acteoside. *Brevundimonas* exhibited a significant positive correlation with Quercetin (*P* < 0.05). *Devosia* showed a significant positive correlation with Apigenin. *Streptomyces* demonstrated a significant positive correlation with Hyperoside (*P* < 0.05). norank_f__Caulobacteraceae and *Pseudomonas* both exhibited significant negative correlations with Echinacoside (*P* < 0.05). *Lactobacillus* showed significant negative correlations with Plantamajoside and Luteolin (*P* < 0.05). For fungi, the dominant microbial groups of endophytic root fungi predominantly belong to the Ascomycota phylum as the primary dominant fungal phylum, followed by Glomeromycota and Basidiomycota, consistent with the previous results on the species composition of dominant fungi communities. *Trichosporiella* exhibited a highly significant positive correlation with Apigenin (*P* < 0.001), while *Cadophora* showed a highly significant positive correlation with Hyperoside (*P* < 0.001). Ceratobasidiaceae_gen_Incertae_sedis showed significant positive correlations with Plantamajoside and Apigenin-7-O-β-glucoside. *Cladosporium* exhibited a significant negative correlation with Apigenin, while *Leptodophora* showed a significant negative correlation with Quercetin (*P* < 0.05).

## 4 Discussion

Medicinal plants are exposed to multiple internal and external factors, which interact to influence the biosynthesis and accumulation of secondary metabolites (Li et al., 2023). Typically, medicinal plants possessing superior qualities and distinctive therapeutic effects are termed authentic medicinal materials (Nottingham et al., 2018). The formation of authentic medicinal materials is closely linked to their specific growing environment, encompassing environmental factors such as temperature, light exposure, altitude, and soil microorganisms (Hou et al., 2025). These factors influence the composition and content of secondary metabolites, collectively determining the quality and efficacy of medicinal plants.

### 4.1 4500 m elevation as a critical ecological threshold: the pivotal role of the soil environment

The influence of altitude, as a key ecological factor, on the accumulation of secondary metabolites in medicinal plants has been demonstrated in numerous high-altitude medicinal plant species (Wu et al., 2025). This study reveals that an elevation of 4500 m is a critical threshold for the ecological adaptation of *Lagotis brevituba*, a boundary primarily driven by significant changes in soil physicochemical properties. Compared to the mid-altitude group (HA, 3500–4500 m), the high-altitude group (EA, >4500 m) exhibited significantly lower levels of soil organic matter (SOM) and available phosphorus (AP), while pH and total nitrogen (TN) levels were higher. According to soil nutrient classification standards, the content of organic matter, available phosphorus, and total nitrogen can serve as proxy indicators of soil nutrient availability. SOM is the primary source of soil nutrients and an important carbon source for microbial metabolism, while AP is a key nutrient that limits the growth of plants and microorganisms. This synergistic pattern of changes in these soil parameters reflects typical high-altitude stress characteristics: Limitations in carbon sources and the bioavailability of phosphorus indicate oligotrophic conditions, while elevated pH reflects a trend toward soil alkalization. This nutrient-poor condition, combined with an alkaline pH (which was significantly higher in the EA group), constitutes the stress characteristics of high-altitude habitats, driving plant-microbe co-adaptive strategies. Tian et al. (2021) indicates that soil bacterial diversity and composition exhibit changes along both surface and deep layers across an elevation gradient. This gradient may alter soil pH, C availability, and C fractions, thereby driving shifts in bacterial diversity and community composition, which resonates with the soil-microbial coupling patterns observed in this study. Similarly, Chen et al. (2022) employed non-targeted metabolomics using UPLC-Q-Exactive-MS coupled with multivariate analysis to distinguish *A. tanguticus* samples from different geographical origins. The results indicate that significant differences exist in secondary metabolites between Tibetan and non-Tibetan regions, with these variations frequently attributable to geographical and environmental factors such as altitude, temperature, and precipitation. Confirming the synergistic regulatory role of elevation and soil factors. Wang Z. et al. (2025) compared Codonopsis grown at high altitudes (2300 m) with that grown at low altitudes (1480 m) and found that triterpenoid compounds accumulated more readily in the former. This was associated with upregulated expression levels of seven key enzyme genes in the triterpenoid biosynthetic pathway. Through a combined metabolomic and transcriptomic analysis, the study confirmed that altitude significantly influences the accumulation of secondary metabolites in medicinal plants. Zhang H. et al. (2025) found that the key bioactive compounds in *Clematis tangutica* (Maxim.) Korsh. accumulate most significantly at elevations between 2500 and 3500 m, indicating that elevation is a key factor driving the accumulation of medicinal compounds in this species. These studies are consistent with the findings of this study. This study clearly anchors the 4500 m elevation threshold to a qualitative change point in soil physicochemical properties, providing a quantifiable soil ecological foundation for understanding the environmental adaptation of alpine medicinal plants.

### 4.2 The screening role of the soil environment on microbial communities

Soil nutrients can directly influence the bioactive constituents of medicinal plants and affect the composition of soil microorganisms (Jiao et al., 2018; Su et al., 2023). Our research indicates that root microbial communities exhibited a higher proportion of dominant phyla alongside reduced diversity, whereas soil microbial communities displayed greater complexity at the phylum level. This aligns with Zhang L. et al. (2025) findings regarding soil microbial diversity. Notably, this study revealed that bacterial community structure exhibited a significant response to altitudinal variation, whereas fungal communities showed no significant differences (Figure 4). This discrepancy may be attributed to the following mechanisms. First, bacteria possess higher metabolic flexibility and environmental adaptability, with community assembly being more directly regulated by factors such as soil pH and nutrient availability. In this study, significant differences in pH, SOM, and AP between the EA and HA groups (Figure 1a) may have preferentially driven the differentiation of bacterial communities. Second, fungal communities generally exhibit greater environmental tolerance and dispersal limitation, with their assembly processes potentially dominated by stochastic factors, resulting in a more delayed response to altitudinal gradients. Furthermore, endophytic fungal communities may form more stable symbiotic relationships with their hosts, buffering the effects of environmental fluctuations. Bahlouli et al. (2025) investigated how altitude influences bacterial communities within the rhizosphere and root-associated microbiome of *V. sylvestris*, and how microbial composition shifts along an altitude gradient. Findings indicate that rhizosphere diversity diminishes with increasing altitude, whereas root-associated diversity increases. Elevation reduced rhizosphere diversity through soil degradation, whereas in the endosphere, it directly boosted diversity by selecting stress-adapted taxa. Altitude plays a significant role in shaping beneficial root-zone microbial communities. This is consistent with the response patterns of root-associated bacterial communities to altitude observed in this study. These findings suggest that bacterial communities serve as more sensitive environmental indicator groups when assessing the microbial ecological functions of high-altitude medicinal plants.

### 4.3 Synergistic effects of the environment and microorganisms on the synthesis of secondary metabolites

The oligotrophic and alkaline stress conditions in high-altitude soils not only reshaped the microbial community structure but also stimulated a significant accumulation of antioxidant secondary metabolites in *Lagotis brevituba*. Significantly elevated levels of β-Sitosterol, Quercetin, and Plantamajoside (*P* < 0.01) in the EA group indicate a positive correlation. Under nutrient-deficient and alkaline stress conditions, plants adapt to adverse environments by upregulating their antioxidant defense systems, thereby increasing the accumulation of phenolic and flavonoid compounds with free radical scavenging activity. Xie et al. (2023) found that moderate drought stress can increase the content of secondary metabolites in the roots of the medicinal plant *Rheum officinale* through two mechanisms: physiological induction and enhanced interactions with beneficial microorganisms. Liang et al. (2025) revealed that in the alpine plant *Meconopsis* (*Meconopsis* spp.), the key enzyme gene MbDDC-3 undergoes functional diversification and positive selection under prolonged high-altitude stress, enhancing the plant’s resistance to bacterial infection, UV radiation, and browsing by promoting the accumulation of alkaloid intermediates. A study by Wang L. et al. (2025) on *Phlomoides rotata* showed that flavonoid biosynthetic pathways were significantly enriched along an altitude gradient of 4300–5000 m, and stress-responsive metabolites such as procyanidin B2 increased significantly with increasing altitude. Based on the findings from these studies on high-altitude medicinal plants, it can be inferred that high-altitude stress not only inhibits growth but also influences the levels of secondary metabolites associated with antioxidant activity and cold tolerance through symbiotic interactions between the rhizosphere soil, functional microbial communities, and the plants themselves, thereby enhancing their medicinal activity.

### 4.4 Key microbial OTUs as mediators of environment-metabolite associations

Numerous studies employing network analysis methods have identified key species across multiple environments, whose effects on community composition and function are independent of their abundance. Investigating the importance of key taxa and key groups for microbial community structure and function is becoming increasingly significant (Banerjee et al., 2018). Wang et al. (2023) investigated the decomposition processes and residue composition dynamics of maize and wheat straw alongside their associated microbiomes. They demonstrated that key microbial groups regulate divergent-convergent trajectories in residue chemistry. Our findings are consistent with this observation. To elucidate the specific roles in the environment-metabolite relationships within microbial communities, this study employed Zi-Pi network topology analysis to identify key OTUs in *Lagotis brevituba*. The results revealed that 19 highly connected key nodes were identified in *Lagotis brevituba*, including 14 bacterial and 5 fungal OTUs, 84% of which belonged to the Proteobacteria and Actinobacteria phyla. The root-associated bacterial soil network revealed that norank_c__MB-A2-108 exhibited significant positive correlations with total nitrogen (TN) and pH, and significant negative correlations with available phosphorus (AP) (*P* < 0.05). Meanwhile, the key node *Aquabacterium* showed significant negative correlations with Luteolin and Acteoside (*P* < 0.05), while *Streptomyces* demonstrated significant positive correlations with Hyperoside (*P* < 0.05). These key taxa occupy “modular hub” or “connector” positions within the co-occurrence network, confirming that key microbial communities play a crucial role in regulating the secondary metabolites of medicinal plants. This provides quantifiable microbiological markers for authenticating “genuine medicinal properties.” Yeoh et al. (2017) study root microbiomes of different plant phyla across a tropical soil chronosequence. They confirm that soil type is the primary determinant of root-associated bacterial communities, but also observe a clear correlation with plant phylogeny and define a core root microbiome at this site. Arbuscular mycorrhizal fungi (AMF) directly influence the production of secondary metabolites by increasing plant biomass, or indirectly affect secondary metabolite production by stimulating biosynthetic pathways (Zhao et al., 2022).

**FIGURE 9:**
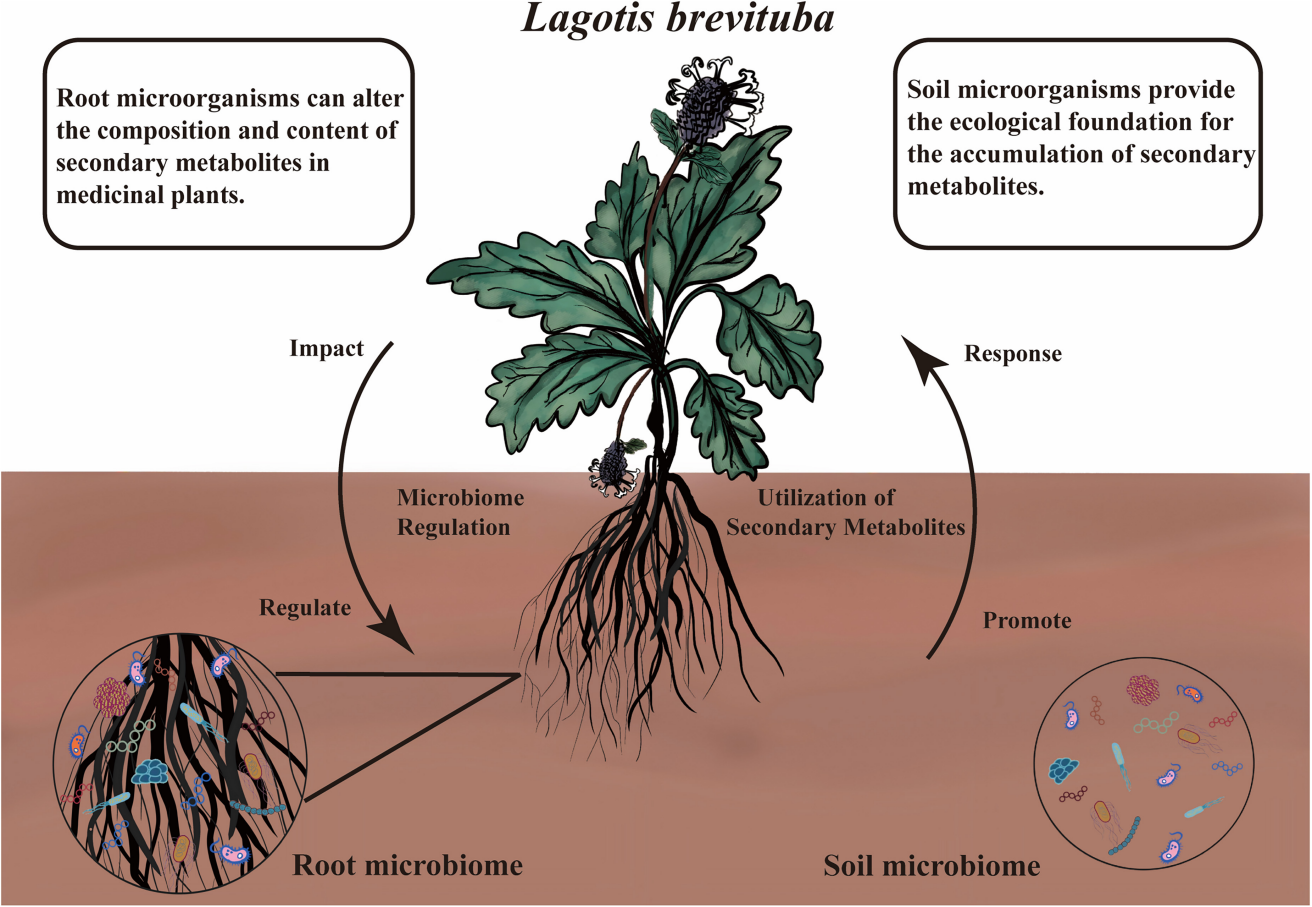
The schematic diagram summarizes the diversity of endophytic microorganisms and rhizosphere soil microorganisms in *Lagotis brevituba* across different production areas, along with their association with 10 key secondary metabolites.

This study innovatively reveals the diversity of root-associated microorganisms and rhizosphere soil microorganisms in *Lagotis brevituba* across different production areas, along with their association with 10 key secondary metabolites. An altitude of 4500 m represents a key threshold where significant differences in *Lagotis brevituba* emerge. With increasing altitude, the content of most secondary metabolites also significantly increases. This suggests that higher altitudes may prompt *Lagotis brevituba* to synthesize more antioxidant-type metabolites, endowing it with higher biological activity or stronger antioxidant capacity to adapt to harsh environments. Furthermore, both root and soil microorganisms influence the composition and content of *Lagotis brevituba*’s secondary metabolites through effects such as influencing, regulating, promoting, and responding. Among these, microbial communities within certain key groups play a crucial role in regulating the plant’s secondary metabolites (Figure 9).

### 4.5 Limitations of the study and future directions

Although extensive exploratory research has been conducted on the active components of *Lagotis brevituba*, yielding a wealth of data and findings, its status as a perennial herbal medicine means that research cycles are relatively long. Furthermore, its quality is influenced by a variety of ecological factors, and the complex interactions among these factors make it difficult to simply elucidate the ecological mechanisms underlying its quality formation. Sample collection for this study covered only parts of Qinghai, Sichuan, and Xizang, and its molecular regulatory mechanisms have not yet been elucidated. Future studies could combine transcriptomics (RNA-seq) and metabolomics technologies to analyze the expression patterns of key enzyme genes (such as PAL, C4H, and DXS) in the secondary metabolic pathways of *Stachys tubulosa* (e.g., the phenylpropanoid pathway and terpenoid synthesis pathway), thereby identifying the molecular switches by which ecological factors regulate the synthesis of active compounds. The limitation of this study lies in the sample size (*n* = 12), While the composite sampling strategy was necessary to capture broad geographic and altitudinal variation across the Qinghai-Xizang Plateau, it inevitably limits the precision of effect size estimates and the generalizability of correlation-based inferences. Future studies with expanded replication and independent validation cohorts are warranted to corroborate these preliminary findings. This study identified key microbial communities, such as *Streptomyces* and *Aquabacterium*, significantly associated with bioactive compounds through network analysis; however, strain isolation, cultivation, and back-cross validation have not yet been conducted. Future work should establish a strain repository of endophytic and rhizosphere microorganisms from *Lagotis brevituba*, verify the regulatory effects of functional strains on host secondary metabolism through a sterile seedling co-culture system, and develop specialized microbial biofertilizers to provide microecological regulation technologies for artificial cultivation.

## 5 Conclusion

This study coupled 16S/ITS high-throughput sequencing with HPLC quantitative analysis to systematically elucidate the diversity of root-associated and rhizosphere soil microorganisms in *Lagotis brevituba* different production areas on the Tibetan Plateau, along with their correlations with ten key secondary metabolites. In high-altitude regions, stressors such as lnutrient-deficient and alkaline stress conditions significantly reduce soil organic matter and available phosphorus content. This environment drives *Lagotis brevituba* to synthesize increased levels of antioxidant metabolites. Microbial community analysis revealed that bacterial community structure exhibited a significant response to changes in elevation, whereas fungal communities showed no significant differences. A joint analysis of the correlation network and Z-Pi values identified 19 key OTUs. *Aquabacterium* exhibited significant negative correlations with Luteolin and Acteoside, while *Streptomyces* showed significant positive correlations with Hyperoside. This study demonstrates that soil environmental factors structure microbial communities, which in turn play a crucial role in regulating the secondary metabolites of medicinal plants. These findings not only fill a gap in understanding the association mechanisms between the microbiome and secondary metabolism in *Lagotis brevituba*, but also provide a universal theoretical framework and technical pathway for the resource conservation, quality enhancement, and sustainable utilization of other rare high-altitude medicinal plants.

## Data Availability

All sequencing data generated or analyzed during this study have been submitted to the NCBI Sequence Read Archive (SRA) database under accession numbers PRJNA1448557 and PRJNA1448718.

## Footnotes

1
http://drive5.com/uparse/

2
http://www.mothur.org/wiki/Calculators
