# Supplementary material for: Analysis of microbial diversity in Lagotis brevituba Maxim. from different production areas on the Qinghai-Xizang Plateau and its correlation with secondary metabolic products
Source: Front Microbiol. 2026 May 5;17:1797784. doi: 10.3389/fmicb.2026.1797784 (PMC13185688; doi:10.3389/fmicb.2026.1797784)
Supplement: Supplementary file 1 [file Table_1.DOC]

**Table 1 Specific sampling information and grouping details for Lagotis brevituba.**

| No. | Altitude(m) | Longitude | Latitude | Group |
| --- | --- | --- | --- | --- |
| LB1 | 3920 | 101°23′38.45″ | 37°21′0.14″ | HA |
| LB2 | 3962 | 98°52′31.41″ | 37°10′32.08″ | HA |
| LB3 | 3566 | 100°16′0.25″ | 38°5′30.59″ | HA |
| LB4 | 4078 | 100°14′23.05″ | 38°0′53.15″ | HA |
| LB5 | 4497 | 99°30′59.70″ | 35°29′54.14″ | HA |
| LB6 | 4364 | 99°18′51.08″ | 35°24′6.10″ | HA |
| LB7 | 4571 | 96°28′21.10″ | 31°57′13.23″ | EA |
| LB8 | 4729 | 95°11′3.44″ | 32°57′49.98″ | EA |
| LB9 | 4660 | 95°52′56.58″ | 32°56′39.14″ | EA |
| LB10 | 4839 | 97°12′44.92″ | 32°34′14.55″ | EA |
| LB11 | 4733 | 98°50′21.73″ | 31°47′10.31″ | EA |
| LB12 | 4629 | 96°40′44.98″ | 31°23′58.55″ | EA |
