# Supplementary material for: Analysis of microbial diversity in Lagotis brevituba Maxim. from different production areas on the Qinghai-Xizang Plateau and its correlation with secondary metabolic products
Source: Front Microbiol. 2026 May 5;17:1797784. doi: 10.3389/fmicb.2026.1797784 (PMC13185688; doi:10.3389/fmicb.2026.1797784)
Supplement: Supplementary file 2 [file Table_2.docx]

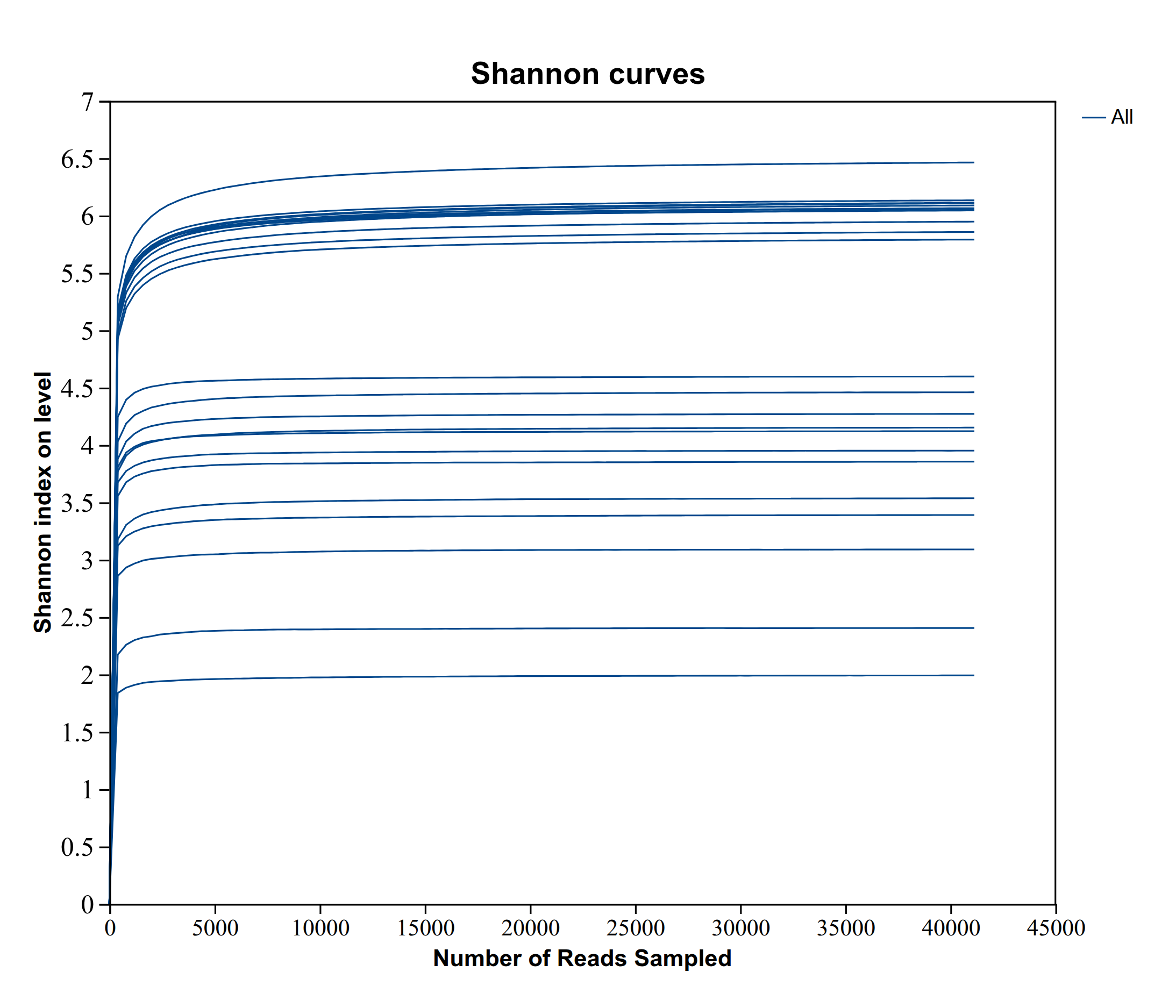


**Figure S1 Bacterial dilution curves at the OTU level are used to assess whether the sequencing depth of each sample is sufficient to reflect the true diversity of the microbial community.**

**
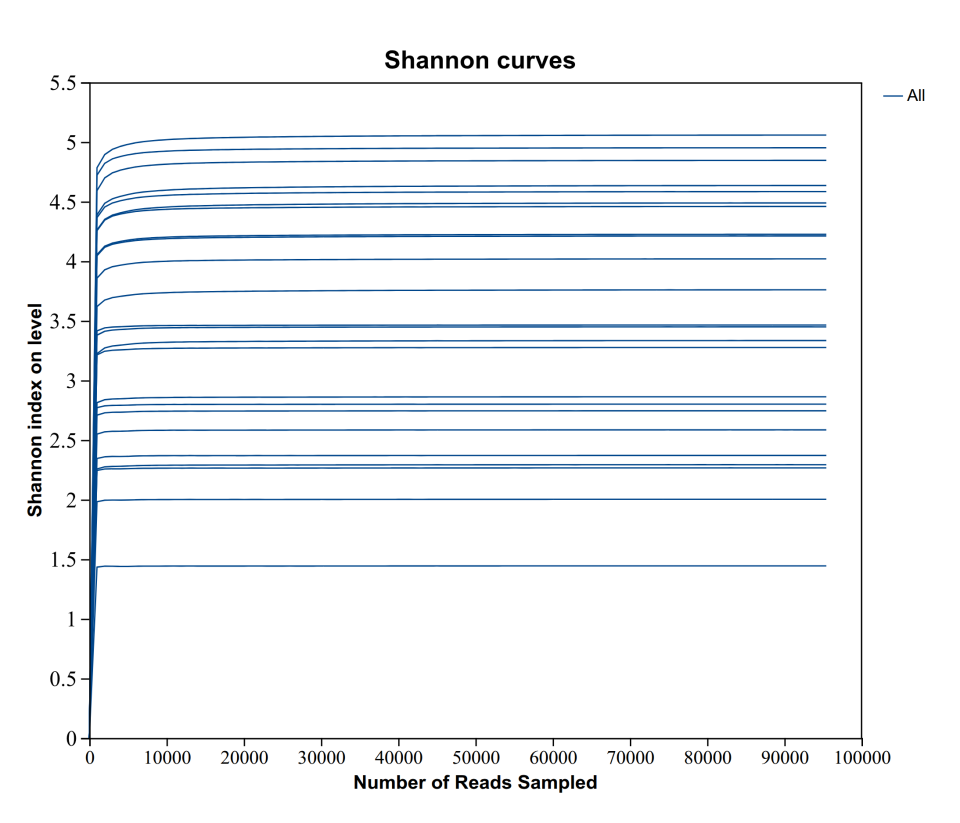
**

**Figure S2 Fungal dilution curves at the OTU level are used to assess whether the sequencing depth of each sample is sufficient to reflect the true diversity of the microbial community.**
